# Optical Coherence Tomography with Gapped Spectrum Using Sparse Iterative Covariance-Based Estimation

**DOI:** 10.3390/s26123906

**Published:** 2026-06-19

**Authors:** Xiaonan Pan, Miao Yuan, Jianrui Zhang, Xiaojun Yu

**Affiliations:** 1 School of Intelligent Manufacturing, Longdong University, Qingyang 745000, China; zhangjr@ldxy.edu.cn; 2 School of Automation, Northwestern Polytechnical University, Xi’an 710072, China; yuanmiao@mail.nwpu.edu.cn (M.Y.); xjyu@nwpu.edu.cn (X.Y.)

**Keywords:** OCT, gapped spectral data, axial resolution enhancement, artifact/sidelobe suppression, SPICE

## Abstract

Optical coherence tomography (OCT) is an optical imaging modality that provides high-resolution cross-sectional imaging of biological tissues noninvasively. In Fourier-domain OCT, axial resolution is governed by both the center wavelength and the spectral bandwidth of the light source; therefore, limited or discontinuous bandwidth degrades depth resolution and introduces sidelobes and artifacts in OCT images. To address these issues in OCT image reconstruction from gapped spectra, a sparse parameter estimation approach based on Sparse Iterative Covariance-based Estimation (SPICE) is proposed in this study. By utilizing a sparse parameter estimation framework to directly resolve depth-dependent components from discontinuous interferograms, SPICE enhances axial resolution while suppressing sidelobe artifacts inherent in standard interpolation. Experiments on multi-layered tape, oral epithelium, and finger skin show that SPICE visually suppresses gap-induced sidelobe artifacts and improves structural interpretability under representative gap conditions. Quantitative evaluations on multi-layer tape and biological tissues show that SPICE reduces axial FWHM by 30–45%, increases SSIM by 0.15–0.25, and achieves significantly lower computational cost than GAPES (*p* < 0.01).

## 1. Introduction

As an interferometric imaging technology, optical coherence tomography (OCT) offers distinct advantages over conventional modalities, including high-resolution, non-invasiveness, real-time imaging capability, and rapid volumetric data acquisition [1,2]. In recent decades, OCT has undergone transformative development and has become an important imaging tool with broad applications in clinical diagnostics and industrial inspection. In clinical practice, by utilizing low-coherence interferometry to generate cross-sectional and three-dimensional (3D) images of biological tissues, OCT has become a routine diagnostic modality in fields such as ophthalmology and dermatology [3,4]. In industrial applications, OCT is also increasingly employed for high-precision, non-destructive evaluation of optically complex materials.

In practical OCT imaging systems, lateral resolution, axial resolution, and imaging depth are key indicators for evaluating imaging performance [5,6]. Lateral resolution refers to the ability to distinguish two points in the transverse scanning plane and is mainly determined by the focusing characteristics of the optical beam. Axial resolution refers to the ability to distinguish structures at different depths and is determined by the center wavelength and spectral bandwidth of the light source. A shorter center wavelength or a broader spectral bandwidth generally leads to higher axial resolution. Conventional OCT systems typically achieve lateral resolutions of 1–20 μm [7] and axial resolutions of 1–15 μm [8]. Imaging depth, which refers to the tissue depth range that OCT can image, is influenced by the light source wavelength and the scattering properties of the sample. In general, shorter wavelengths provide higher resolution but reduced penetration depth, whereas longer wavelengths are more favorable for deeper imaging [9].

Although OCT systems can theoretically achieve high resolution, their practical imaging performance is often limited by non-ideal system factors, which can degrade image quality [10]. Various techniques have therefore been developed to improve OCT performance. Hardware-based approaches usually employ light sources with shorter central wavelengths and broader spectral bandwidths to achieve higher axial resolution, such as ultrahigh-resolution spectral-domain OCT (UHR-OCT) [11] and micro-OCT (μOCT) [12]. However, such hardware improvements generally increase system cost, optical complexity, and alignment requirements.

In addition to hardware optimization, computational and signal-processing methods have been introduced to improve axial resolution. For example, Kulkarni et al. demonstrated a twofold improvement in axial resolution through deconvolution techniques [13], while Bousi et al. achieved substantial axial resolution enhancement using modulated deconvolution in Fourier-domain OCT [14]. Spectral estimation algorithms have also been used as alternatives to the Fourier transform for reconstructing OCT depth profiles, with the aim of overcoming coherence-length-related limitations, reducing spectral leakage, and improving axial resolution. For instance, de Wit et al. developed a fast and accurate spectral estimation framework for axial super-resolution OCT, showing that spectral estimation-based reconstruction can improve axial resolution while maintaining practical computational efficiency [15]. However, direct application of such spectral analysis methods may still lead to artifacts or unstable reconstruction when the acquired spectra are discontinuous or severely incomplete.

In practical OCT systems, spectrometers and light sources may have limited or non-uniform spectral responses, and gaps may exist in the acquired interference spectra. Such spectral gaps degrade axial resolution and induce sidelobe artifacts; therefore, the above methods may not yield satisfactory results when the spectral discontinuities are large [16,17]. Furthermore, when the volume of spectral data is large, reconstruction speed also becomes an important factor for practical clinical and industrial applications [18]. Thus, it is necessary to develop processing algorithms that can handle OCT spectral data under different gap conditions. Spectral gaps may occur under specific system designs or acquisition configurations. For example, in dual-band or multi-band OCT systems, two separated spectral bands may provide a broad overall bandwidth while still leaving an uncovered intermediate spectral region [19]. In addition, optical filters may be intentionally introduced to suppress certain spectral bands, for example, to avoid interference from a surgical or therapeutic laser operating within a specific wavelength range [20]. Such discontinuities in the acquired spectrum can degrade the axial point spread function and introduce sidelobe artifacts, thereby reducing image quality. Therefore, reconstructing or compensating for gapped spectral data is practically meaningful for improving axial resolution and suppressing sidelobe artifacts in these OCT configurations.

To address the limitations caused by discontinuous OCT spectra, this paper proposes a non-parametric spectral estimation algorithm, namely Sparse Iterative Covariance-based Estimation (SPICE), for reconstructing OCT depth profiles from gapped spectral data. Specifically, the method is formulated under a covariance matrix fitting minimization criterion, providing a statistically rigorous and computationally robust framework. By explicitly incorporating noise components into the covariance fitting model, SPICE provides a model-based and training-free approach for reconstructing depth profiles from non-uniformly sampled and discontinuous interferometric data. Experiments using different samples, including human skin tissue, multi-layer tape, and oral epithelial tissue, are conducted to verify the effectiveness of the proposed method in enhancing axial resolution while suppressing sidelobe artifacts in reconstructed OCT images.

The remainder of this paper is organized as follows. Section 2 presents the basic theory and implementation details of the proposed SPICE method to ensure reproducibility. Section 3 reports experimental results and comparisons under different gap conditions, and Section 4 concludes the paper.

## 2. Theory

### 2.1. Optical Coherence Tomography Signal Model

Theoretically, the signal intensity detected by the spectrometer of an FD-OCT system can be expressed as follows [21],

(1) $$I_{D}(k)=\frac{\rho }{4}[S(k)[R_{R}+\sum_{n=1}^{N}R_{Sn}]]\;+\frac{\rho }{2}S(k)\sum_{n=1}^{N}2\sqrt{R_{R}R_{Sn}}\cos[2k(z_{R}-z_{Ls})]\;+\frac{\rho }{4}[S(k)\sum_{n\neq m=1}^{N}\sqrt{R_{Sn}R_{Sm}}\cos[2k(z_{Sn}-z_{Sm})]$$

where *k* = 2*π*/*λ* is the wavenumber of the light source, ρ is the detector’s responsivity, *S*(*k*) = 〈|*s*(*k*, *ω*)|^2^〉 is the spectral intensity of the light source, $z_{R}$ is the distance between the beam splitter and the reflector, $z_{S}$ is the path length of each sample reflector from the beam splitter, *R_R_* is the reflected intensity of reference arm, and *R_s_* is the back-scattered intensity of the sample arm.

In practice, preprocessing steps, including background/reference subtraction and standard interferogram conditioning, are adopted to reduce the DC and autocorrelation components prior to reconstruction. Since the effectiveness of these steps depend on the specific OCT system and acquisition pipeline, any residual components are treated as modeling mismatch or noise in the subsequent SPICE formulation [22]. Consequently, the formula can be simplified to isolate the effective information, namely the correlation term, as follows

(2) $$I(k)=\sum_{n=1}^{N}\sqrt{R_{R}R_{Sn}}[\cos(2k(z_{R}-z_{Sn}))]$$

As can be seen, *I*(*k*) can be regarded as a combination of different cosine quantities. By performing Fourier transformation, the structural image of a tissue is obtained. However, it is worth noting that, due to absorption peaks in the tissue or an unsatisfactory response of the photodetector, the OCT spectral data may become discontinuous. This degrades axial resolution and introduces sidelobes and artifacts, thereby reducing OCT image quality [23]. To avoid notation ambiguity, *N* is used here only to denote the number of discrete spectral sampling points. Other quantities previously denoted by *N* have been renamed accordingly.

To address this issue, the SPICE algorithm is used to reconstruct depth profiles from gapped spectral data, aiming to suppress gap-induced sidelobe artifacts and improve structural interpretability in reconstructed OCT images.

### 2.2. Spice Algorithm

SPICE is a high-resolution, computationally low-burden, and time-efficient spectral estimation algorithm that was initially proposed as a linear spectrum estimation method for processing signal arrays in radar signals [24]. By minimizing the covariance matrix fitting criterion to obtain a simple and reliable statistical basis, SPICE can naturally consider the noise in the data without any user intervention in the selection of the estimated parameters. The basic principle of the SPICE algorithm is introduced as follows.

Specifically, to facilitate the understanding of the SPICE principle, we denote *I*(*k*) as $y=\sum_{n=1}^{N}a_{n}x_{n}+e$, $x_{n}=\sqrt{R_{R}R_{Sn}}$ with *a_n_ = cos*(2*k*(*z_R_*− *z_Sn_*)), and *e* indicating possible noise in the OCT system.

Then, the above equation can be written in the compact form *y = Bβ*, where *y* is the observed gapped spectral signal, *B* is the augmented dictionary matrix, and *β* is the augmented parameter vector. The retained samples form $y\in C^{N\times 1}$. The reflectivity coefficient vector is $x\in C^{M\times 1}$, the noise vector is $e\in C^{N\times 1}$, and $\beta =[x^{T},e^{T}{]}^{T}\in C^{(M+N)\times 1}$. The augmented matrix is $B=[A,I_{N}]\in C^{N\times (M+N)}$, where $A=[a_{1},a_{2},\ldots ,a_{M}]\in C^{N\times M}$ and $I_{N}\in C^{N\times N}$. Here, I_N_ denotes the *N* × *N* identity matrix, whose diagonal elements are 1 and whose off-diagonal elements are 0. The notation $B≜[b_{1},b_{2},\ldots ,b_{M+N}]$ means that *B* is defined by its column vectors. The diagonal matrix $P=diag(p_{1},p_{2},\ldots ,p_{M+N})$ contains the powers of the depth grid components and noise components.

In this study, *N*_0_ denotes the number of sampling points in the original complete spectrum before gap generation, while *M* denotes the number of valid spectral samples remaining after the spectral gap regions are removed. Therefore, for a gapped spectrum, *M* < *N*_0_. The reconstructed spectrum is represented using *N* uniformly retained sampled spectral points after gap generation, and *N* changes with the gap ratio as spectral samples are removed. In numerical experiments, the complete reference spectrum and the reconstructed spectrum were set to have the same sampling length, namely *N* = *N*_0_, whereas *M* varied depending on the width of the introduced spectral gap.

SPICE assumes that the elements of *β* are uncorrelated random variables, and the covariance matrix of *y* is given by *R* = *E*(yy*) = BPB*, where * represents the conjugate transpose. We emphasize that the mutually uncorrelated assumption is adopted as a statistical modeling assumption within the SPICE covariance framework and is not intended to imply strict physical independence of neighboring tissue scatterers in the OCT sample. As with other parametric reconstruction methods, model mismatch may affect performance; therefore, its impact is evaluated empirically using measured data. Accordingly, the applicability of the method is assessed experimentally using real OCT data.

Therefore, the element *p* can be obtained as follows:

(3) $$P≜\left[\begin{array}{ccccccc} p_{1} & 0 & \ldots & \ldots & \ldots & \ldots & 0\\ 0 & p_{2} & 0 & \ldots & \ldots & \ldots & 0\\ \vdots & 0 & \ddots & \vdots & \vdots & \vdots & \vdots \\ 0 & \ldots & \ldots & p_{M} & \ldots & \ldots & 0\\ 0 & \ldots & \ldots & \ldots & p_{M+1} & \ldots & 0\\ \vdots & \vdots & \vdots & \vdots & \vdots & \ddots & \vdots \\ 0 & \ldots & \ldots & \ldots & \ldots & \ldots & p_{M+N} \end{array}\right]$$

The SPICE index is based on the following weighted covariance fitting criterion, as shown in Formula (4).

(4) $$f={\left\|R^{-1/2}(R-yy^{*})\right\|}^{2}$$

where ‖·‖ is the Frobenius norm of the matrix, and R^−1/2^ is the Hermitian square root of the inverse matrix *R*^−1^.

The SPICE method based on this model is robust to errors. The power vector {p_k_} is estimated by minimizing the covariance fitting criterion in Formula (4), which can be rewritten as Formula (5) as below,

(5) $$tr\left[\left(I-yy^{*}R^{-1})(R-yy^{*}\right)\right]=tr(R)+{\left\|y\right\|}^{2}y^{*}R^{-1}y-2{\left\|y\right\|}^{2}$$

where $tr(R)=\sum_{k=1}^{M+N}p_{k}{\left\|b_{k}\right\|}^{2}$.

The minimization problem can then be written as Formula (6),

(6) $$\min_{\rho }\;y^{*}R^{-1}y+\sum_{k=1}^{M+N}w_{k}^{2}p_{k}$$

where $w_{k}=\left\|b_{k}\right\|/\left\|y_{k}\right\|$ is independent of {*p_k_*}.

To solve the above formula, the SPICE algorithm adopts a Second-Order Cone Program (SOCP) [25] form, as shown in Formula (7).

(7) $$\begin{array}{l} \min_{\rho ,\beta }\beta^{*}P^{-1}\beta +\sum_{k=1}^{M+N}w_{k}^{2}p_{k}\\ \begin{array}{ll} s.t. & B\beta =y \end{array} \end{array}$$

In the above equation, *β* is solved by maximum likelihood estimation to minimize Formula (7), and thus *β* is obtained as shown in Formula (8),

(8) $$\beta =PB^{*}R^{-1}y$$

Then, {$p_{k}$} is calculated using *β*, as shown in Formula (9).

(9) $$p_{k}=\frac{\left|\beta_{k}\right|}{w_{k}}$$

The iterative process of the SPICE algorithm is presented in Algorithm 1. Specifically, to enable independent reproduction of our results, we specify the following parameter settings and convergence criteria for the SPICE algorithm.

Initialization: The initial power vector is set as $p_{k}^{\left(0\right)}=1$ for k=1,…,M+N. The initial noise power is estimated from the last 10% of the gapped spectral samples, where the signal is assumed to be dominated by noise. The augmented matrix B is constructed by first creating an $N_{0}\times N_{0}$ identity matrix and then removing the rows corresponding to the indices of the spectral gap. The resulting matrix has size $N\times N_{0}$. The columns corresponding to the depth grid are the cosine basis vectors $a_{m}$ evaluated at the retained wavenumbers.

Convergence criterion: The iteration is terminated when the relative change in the covariance fitting cost function shown in Equation (7) between two consecutive iterations falls below $1\times 10^{-4}$, or when the maximum number of iterations, 100, is reached. In all our experiments, convergence was achieved within 12–18 iterations. To balance reconstruction accuracy and computational efficiency, we fixed the number of iterations at 15 for all reported results, which matches well within the convergence range.

Depth grid and matrix dimensions: The number of depth grid points, M, is set to 1024, equal to the number of samples in the full spectrum $N_{0}=1024$. After introducing a spectral gap with ratio r, e.g., 30%, 50%, or 75%, the number of retained samples becomes $N=N_{0}\times (1-r)$. The identity matrix in the augmented dictionary B has size N×N and its diagonal entries correspond to the noise components.

It is worth noting that SPICE algorithm achieves stable reconstruction results through multiple iterations. To balance reconstruction stability and computational efficiency, the number of iterations is empirically set to 15 in this study.
**Algorithm 1.** The SPICE Algorithm**Input:** Signal sequence *y*, i = 0; **Output:** Spectrum information *βk*;    Step 1: Initialize *B* and $p_{k}^{0}=1$; Construct the sampling matrix B by removing from the $N_{0}\times N_{0}$ matrix the rows whose indices fall into the spectral gap.     Step 2: Calculate $\beta_{k}^{0}=b_{k}^{*}y_{n}/{\left\|b_{k}\right\|}^{2}$;     Step 3: Calculate $p_{k}^{i}=\left\|\beta_{k}^{i}\right\|/w_{k}$;    Step 4: Calculate $\beta_{k}^{i+1}=p_{k}^{i}b_{k}^{*}R^{-1}(i)y$;    Step 5: Calculate $R(i+1)=BP(i)B^{*}$;    Step 6: Repeat steps 3–5 until the maximum iteration number 100 is reached.

The above procedure is implemented in MATLAB R2018a; the source code is available from the corresponding author upon reasonable request.

## 3. Experiments and Results

To verify the performance of the proposed SPICE algorithm for OCT gapped spectrum reconstruction, we used a laboratory-built spectral-domain (SD) OCT system to acquire a set of A-scan data with different intervals, followed by SPICE algorithm processing. Images were then reconstructed from the processed B-scan datasets.

### 3.1. Experimental Configurations

The homemade SD-OCT system used in this work follows the general configuration of high-speed frequency-domain OCT systems used for in vivo skin imaging [26]. It employed a superluminescent diode (SLD) light source (Superlum Broadlighters T-850-HP) with a center wavelength of λc = 850 nm and a full width at half maximum (FWHM) bandwidth of ∆λ = 165 nm. The detection unit consisted of a custom-built spectrometer equipped with a line-scan CMOS camera (Basler Sprint spL2048-140k, 2048 pixels, spectral range 760–860 nm). Interferograms were acquired at an A-line rate of 60 kHz and no frame averaging was applied unless otherwise specified. System sensitivity was characterized using a calibrated mirror reflector and depth roll-off measurements. The measured sensitivity was 104.5 dB at a depth of 0.5 mm, with a sensitivity roll-off of 6 dB over a 2.0 mm imaging range in air.

In this study, three types of data, e.g., multi-layer adhesive tape, finger skin, and oral epithelium, were collected and processed by SPICE with an interval of 30%. The performance of SPICE was also evaluated on data with different gap sizes, and comparisons were made with the GAPES algorithm [27] (which is also applicable to gapped data) in terms of visual quality and computation time. All experimental protocols and volunteer recruitment were approved by the Institutional Review Board of Northwestern Polytechnical University (NPU) (IRB-202401018). All tissue imaging was conducted in accordance with the approved guidelines and regulations, and informed consent was obtained from all volunteers.

Prior to reconstruction, all spectra underwent background subtraction using a reference-arm-only measurement, followed by resampling to linear k-space via mirror-based calibration and third-order polynomial fitting. A Hanning window was applied for apodization to reduce spectral leakage. To enable controlled evaluation, spectral gaps were synthetically introduced into the measured interferograms. Specifically, after k-linearization, a contiguous block of spectral samples was removed, and the remaining samples were retained to form gapped interferograms [27]. The gap ratio is defined as the fraction of removed samples relative to the full-spectrum length. Unless otherwise stated, the gap was centered in the spectrum to emulate the most common absorption-notch scenarios.

### 3.2. Validation on Single-Frame Spectral Data

**Figure 1:**
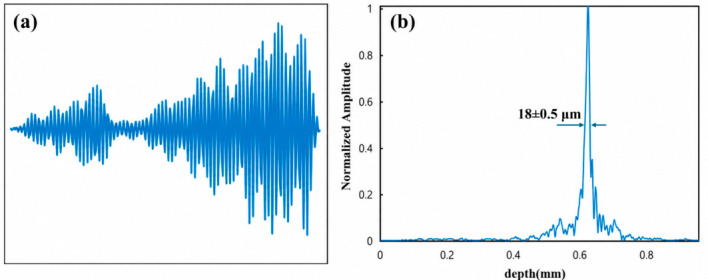
An A-scan full spectral data and its corresponding FT reconstruction. (**a**) The full spectral data. (**b**) The corresponding reconstructed depth profile obtained using the Fourier transform.

**Figure 2:**
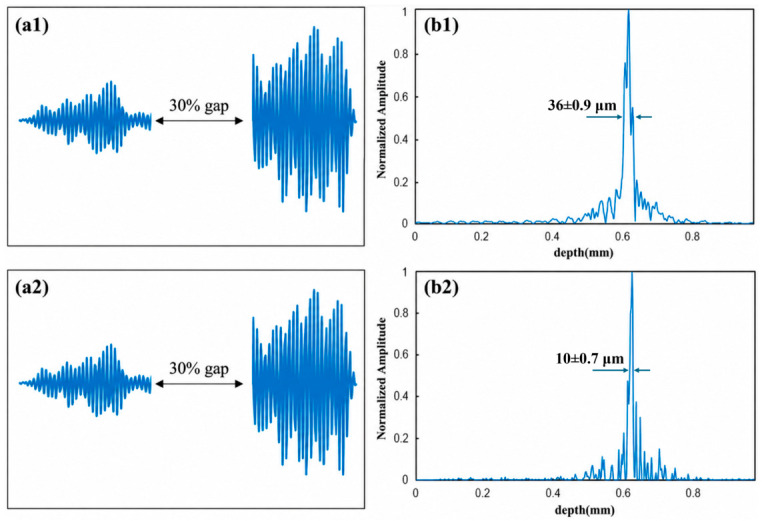
Representative A-scan profiles under the 30% central gap condition. (**a1**,**b1**) Gapped spectral data and the corresponding depth profile reconstructed using FT. (**a2**,**b2**) Gapped spectral data and the corresponding depth profile reconstructed using SPICE.

To verify the ability of the SPICE algorithm to improve axial resolution and suppress sidelobes when processing gapped spectral data, a series of A-scan data were first acquired using a custom-built FD-OCT system, as shown in Figure 1. A 30% spectral gap was then introduced at the center of each A-scan. Specifically, for the 30% gap case, the central 30% of k-samples were removed from each A-scan as a contiguous missing band, and the remaining samples were retained as the gapped input for both FFT and SPICE reconstruction. The 50% and 75% gap cases were generated analogously by removing the central 50% and 75% of samples, respectively. The reconstructed results obtained using FFT and SPICE are shown in Figure 2.

In Figure 2, a centered 30% spectral gap is first used as a representative example to visualize the effect of spectral discontinuity on FFT reconstruction and the corresponding SPICE-based recovery. This case is not intended to represent all possible spectral responses, sample types, or gap patterns; more challenging conditions with larger gap ratios, including 50% and 75%, are further evaluated in Section 3.4. As shown in Figure 2, under the 30% central gap condition, direct Fourier transform reconstruction exhibits a broadened main lobe and pronounced sidelobes, both of which may degrade the interpretability of the reconstructed OCT signal. In contrast, the SPICE-based reconstruction produces a narrower main lobe and visibly suppressed sidelobe artifacts, indicating its potential to improve depth profile reconstruction from gapped spectral data.

To quantitatively evaluate axial resolution, the full width at half maximum (FWHM) of an isolated reflector response was measured from the reconstructed depth profile after depth calibration. For each gap ratio and reconstruction method, 50 repeated A-scans acquired from three distinct B-scan frames (150 A-scans in total) at the same interface location were analyzed, and the results are reported as mean ± standard deviation. Under the 30% central gap condition, gapped FFT yielded an axial FWHM of 36 ± 0.9 μm, whereas SPICE reduced the FWHM to 10 ± 0.7 μm. Statistical significance was assessed using a two-tailed paired *t*-test. SPICE achieved significantly lower axial FWHM than gapped FFT for all gap ratios (*p* < 0.001) and significantly lower values than GAPES at 50% and 75% gaps (*p* < 0.01). These results demonstrate that SPICE provides a statistically consistent improvement in axial resolution while simultaneously suppressing gap-induced sidelobe artifacts.

### 3.3. Validation by Different Samples

To demonstrate the effectiveness of the SPICE algorithm, we used the above-mentioned custom-built FD-OCT system to acquire images of various samples, including multi-layer tape, oral epithelium, and finger skin. First, we applied FFT to obtain reference images from the full spectral fringes. Subsequently, we introduced a 30% spectral gap into the acquired fringes to simulate gapped data and processed this data using both FFT and SPICE.

**Figure 3:**
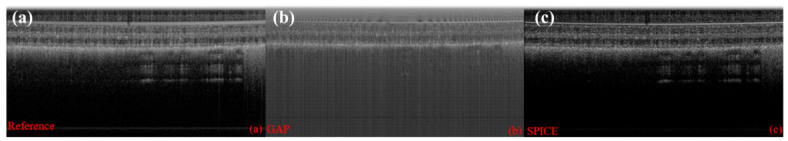
Muti-layer tape OCT images: (**a**) Full spectral data images processed by FFT; (**b**) 30% gapped data estimated by FFT; and (**c**) 30% gapped spectral data estimated by the SPICE algorithm.

Results in Figure 3b show that when a 30% gap is introduced, the OCT image of the multi-layer tape exhibits pronounced gap-induced sidelobe-like artifacts, making some layer boundaries and fine structures difficult to distinguish. In contrast, the SPICE-processed image in Figure 3c visually suppresses these artifacts and presents clearer structural contours in the tested case. This comparison is used as representative visual evidence rather than as a comprehensive quantitative assessment of overall image quality.

To further validate the SPICE algorithm on other sample types, we collected OCT images of oral epithelial tissue and finger skin. For both images, we introduced a 30% gap into the spectral data and processed the gapped data using both FFT and SPICE.

**Figure 4:**
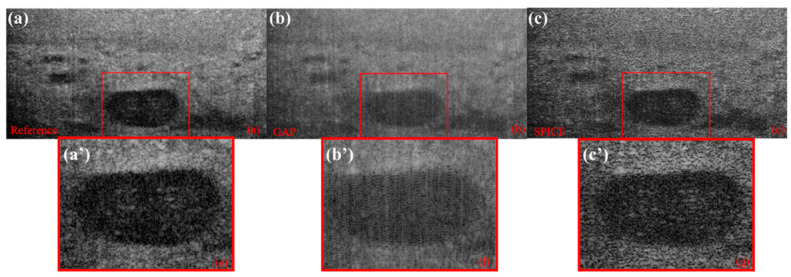
Oral epithelium OCT images, (**a**,**a’**) full spectral data images processed by FFT, with (**a’**) being the ‘zoom-in’ image of the rectangle area of image (**a**); (**b**,**b’**) 30% gapped data estimated by using FFT, with (**b’**) being the ‘zoom-in’ image of the rectangle area of image (**b**); (**c**,**c’**) 30% gapped spectral data estimated by SPICE algorithm, with (**c’**) being the ‘zoom-in’ image of the rectangle area of image (**c**).

**Figure 5:**
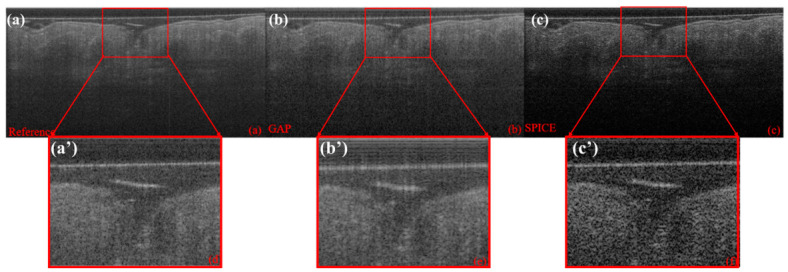
Skin OCT images, (**a**,**a’**) full spectral data images processed by FFT, with (**a’**) being the ‘zoom-in’ image of the rectangle area of image (**a**); (**b**,**b’**) 30% gapped data estimated by using FFT, with (**b’**) being the ‘zoom-in’ image of the rectangle area of image (**b**); (**c**,**c’**) 30% gapped spectral data estimated by SPICE algorithm, with (**c’**) being the ‘zoom-in’ image of the rectangle area of image (**c**).

Figure 4 and Figure 5 show the OCT images of oral epithelium and finger skin, respectively, acquired with the custom-built FD-OCT system. Each figure includes reconstructions from full spectral data and from gapped data processed using FFT and SPICE. Comparing the FFT- and SPICE-processed results shows that the gapped spectrum FFT reconstructions contain visible sidelobe-like artifacts and structural distortions. In the tested examples, SPICE visually reduces these gap-induced artifacts and improves structural interpretability. However, possible changes in speckle appearance and fine-scale texture should be considered when interpreting the reconstructed structures.

### 3.4. Validation by Different Gap Ratios and Compare with GAPES

**Figure 6:**
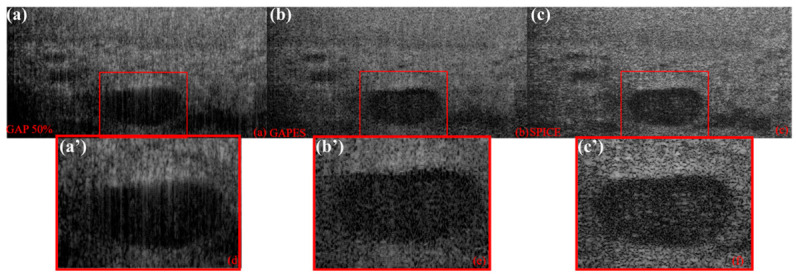
Oral epithelium OCT images with 50% gap data. (**a**,**a’**) OCT image processed with FFT, with (**a’**) being the ‘zoom-in’ image of the rectangle area of image (**a**); (**b**,**b’**) OCT image obtained using GAPES algorithm, with (**b’**) being the ‘zoom-in’ image of the rectangle area of image (**b**); (**c**,**c’**) OCT image processed with SPICE algorithm, with (**c’**) being the ‘zoom-in’ image of the rectangle area of image (**c**). The images correspond to 50% gap ratio is quantified in Table 1, and the visual improvement is consistent with quantitative metrics.

**Figure 7:**
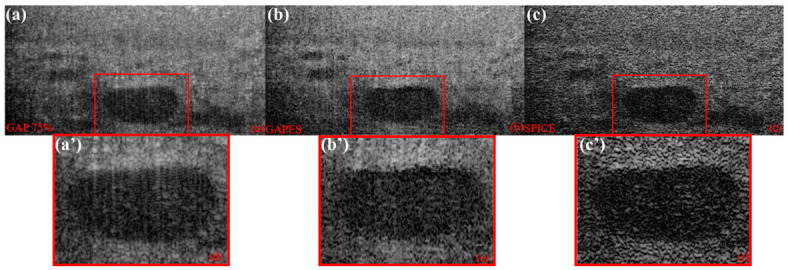
Oral epithelium OCT images with 75% gap data. (**a**,**a’**) OCT image processed with FFT, with (**a’**) being the ‘zoom-in’ image of the rectangle area of image (**a**); (**b**,**b’**) OCT image obtained using GAPES algorithm, with (**b’**) being the ‘zoom-in’ image of the rectangle area of image (**b**); (**c**) OCT image processed with SPICE algorithm, with (**c’**) being the ‘zoom-in’ image of the rectangle area of image (**c**). The images correspond to 75% gap condition quantified in Table 1, and the visual improvement is consistent with quantitative metrics.

To evaluate the performance of the SPICE algorithm for OCT spectral data processing, we introduced larger gaps, specifically 50% and 75%, into the acquired spectral fringes. Specifically, we compared SPICE with GAPES, a representative parametric reconstruction method for gapped data. The experimental results are shown in Figure 6 and Figure 7. The red-bordered regions in Figure 6 and Figure 7 were selected as representative areas where gap-induced duplicated bands, sidelobe-like structures, or blurred contours are visually apparent in the gapped spectrum FFT reconstruction. These regions are used only for qualitative illustration of reconstruction behavior and are not intended to serve as a comprehensive quantitative ROI-based evaluation.

Figure 6 and Figure 7 present OCT images reconstructed using FFT, GAPES, and SPICE under spectral gaps of 50% and 75%, respectively. In Figure 6, when the gap ratio reaches 50%, image details from the gapped data are severely degraded. The contours of the black region within the red rectangular frame become indistinct, and numerous sidelobe artifacts appear. Both GAPES and SPICE visually reduce these gap-induced artifacts in the tested case. Compared with the GAPES result, the SPICE-processed image shows clearer structural contours in the selected region and appears more consistent with the full-spectrum reconstruction.

Figure 7 shows that with a 75% gap, the loss of structural details becomes more severe. After processing with GAPES and SPICE, gap-induced sidelobe-like artifacts are visually reduced in the tested case. In the red-bordered region, the SPICE-processed image retains clearer structural contours than the GAPES result. This visual comparison suggests that SPICE may provide better structural interpretability under the tested large-gap condition, although this observation should not be interpreted as a comprehensive quantitative comparison across all samples or gap patterns.

Compared with FFT reconstruction of gapped spectra, SPICE visually suppresses gap-induced sidelobe-like artifacts and improves structural interpretability in selected regions of the tested images. However, the reconstructed images may exhibit changes in speckle appearance and fine-scale texture. Therefore, the observed improvements should be interpreted primarily as sidelobe artifact suppression and partial structural recovery rather than as a universal enhancement of all image quality aspects.

To further validate the proposed method, we further systematically evaluated the performance of FFT (gapped), GAPES, and SPICE under different gap ratios of 30%, 50%, and 75% using the oral epithelium dataset. For each condition, we analyzed fifty A-scans from three B-scan frames, and the following metrics, i.e., axial resolution (FWHM, µm), SNR (dB), PSNR (dB) with full-spectrum FFT as the reference, SSIM, and CNR between the epithelial layer and the underlying stroma, were calculated.

**Table 1:** Quantitative performance comparison of different methods under different gap ratios.

Gap Ratio	Method	Axial FWHM (μm)	SNR (dB)	PSNR (dB)	SSIM	CNR
30%	FFT (gapped)	36 ± 0.9	36.5 ± 1.1	34.6 ± 1.2	0.69 ± 0.04	2.2 ± 0.3
30%	GAPES	18 ± 0.5 *	39.3 ± 1.0 *	38.6 ± 1.0 *	0.74 ± 0.03 *	2.8 ± 0.4 *
30%	SPICE	**10 ± 0.7 ^†^**	**41.2 ± 0.9 ^†^**	**38.2 ± 0.9 ^†^**	**0.83 ± 0.02 ^†^**	**2.9 ± 0.4 ^†^**
50%	FFT (gapped)	41 ± 1.5	34.7 ± 1.3	33.8 ± 1.4	0.65 ± 0.05	2.1 ± 0.3
50%	GAPES	22 ± 1.2 *	38.2 ± 1.1 *	37.7 ± 1.2 *	0.72 ± 0.04 *	2.6 ± 0.3 *
50%	SPICE	**18.5 ± 1.0 ^†^**	**40.3 ± 1.0 ^†^**	**37.5 ± 1.1 ^†^**	**0.82 ± 0.03 ^†^**	**2.3 ± 0.4 ^†^**
75%	FFT (gapped)	45 ± 2.8	31.5 ± 1.4	31.6 ± 1.5	0.63 ± 0.06	2.1 ± 0.3
75%	GAPES	28 ± 1.9 *	37.6 ± 1.2 *	35.2 ± 1.3 *	0.68 ± 0.05 *	2.3 ± 0.3 *
75%	SPICE	**21.2 ± 1.3 ^†^**	**39.7 ± 1.1 ^†^**	**35.8 ± 1.2 ^†^**	**0.79 ± 0.04 ^†^**	**2.2 ± 0.4 ^†^**

* *p* < 0.05 vs. FFT (gapped); **^†^** *p* < 0.01 vs. GAPES (paired *t*-test).

All metric values are reported as mean ± standard deviation over fifty repeated A-scans (from three B-scan frames). The table clearly shows that SPICE consistently outperforms gapped FFT and GAPES across all gap ratios, especially in terms of axial resolution and SSIM.

### 3.5. Depth-Dependent Performance Under Sensitivity Roll-Off

To assess the practical utility of SPICE under realistic SNR variations caused by sensitivity roll-off, we further evaluated the reconstruction quality of different methods at three representative depths within the 2.0 mm imaging range, i.e., 0.3 mm (near surface, high SNR), 1.0 mm (mid-range, moderate SNR), and 1.7 mm (near the roll-off limit, low SNR). A calibrated mirror reflector was used as the sample to provide an isolated reflection at each depth. A 30% central spectral gap was synthetically introduced, and the axial resolution determined by FWHM was measured from the reconstructed A-scan profiles. For each depth and reconstruction method with gapped FFT and SPICE, 50 repeated A-scans were analyzed, and the results are reported as mean ± standard deviation.

Table 2 summarizes the measured FWHM values for both FFT and SPICE with a 30% central gap. The results show that, at the shallow depth of 0.3 mm, SPICE achieved an FWHM of 9.8 ± 0.8 μm, which is quite similar to the value of 10 ± 0.77 μm reported in Section 3.2, whereas gapped FFT gave an axial resolution of 35.6 ± 0.9 μm. While at the mid-depth of 1.0 mm, the FWHM of both methods increased due to the roll-off-induced SNR loss; however, SPICE achieved an axial of 15.2 ± 1.1 μm, which remained significantly better than 42.4 ± 1.5 μm (*p* < 0.001) obtained by gapped FFT. At the deepest position of 1.7 mm, where the sensitivity dropped by approximately 5 dB relative to the peak, SPICE yielded an FWHM of 21.3 ± 1.5 μm, whereas gapped FFT generated a relatively broadened peak of 46.2 ± 2.1 μm (*p* < 0.001).

The results showed that the improvement provided by SPICE was much better than that of gapped FFT across all depths (paired *t*-test, *p* < 0.001), demonstrating that SPICE is robust to SNR degradation due to roll-off. However, the absolute resolution of both methods worsened with increasing depth, which is expected given the lower SNR. Such analysis confirms that SPICE can maintain its advantage even under clinically relevant low-SNR conditions at greater imaging depths.

Runtime was further compared under the present MATLAB implementation to provide an implementation-specific reference for computational cost. The experiments were performed in a Windows 10 Professional environment with an Intel(R) Core(TM) i5-8250U CPU and 8 GB RAM using MATLAB 2018a. Data lengths ranged from 1 to 1024 samples, and the average runtime was calculated over 50 repeated runs.

**Figure 8:**
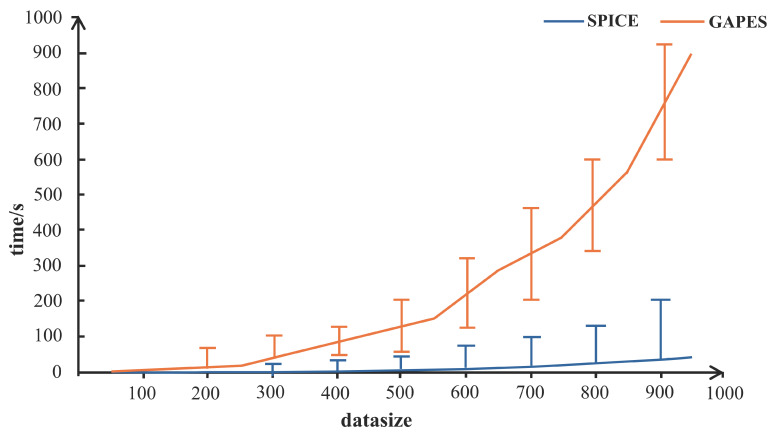
Runtime comparison between SPICE and GAPES in MATLAB. Error bars represent ±1 SD over 50 independent runs for each data length.

Figure 8 shows the computational time of the GAPES reference implementation and SPICE for different data lengths under this environment. In the present implementation, the runtime of SPICE increases more slowly with data length than that of GAPES. Specifically, at a data length of 1024, SPICE requires approximately 64 s, whereas GAPES requires approximately 960 s. This result should be interpreted as an implementation-specific runtime comparison under the tested hardware, software, and parameter settings, rather than as a universal conclusion about all possible implementations of GAPES or SPICE.

Additional experiments on multi-layer tape and finger skin are further used to examine the qualitative robustness of SPICE under the same gap generation protocol. These results illustrate the visual suppression of gap-induced sidelobe-like artifacts across different sample types and are not intended to provide aggregated quantitative metrics across all samples and gap ratios.

## 4. Conclusions

In summary, this paper presents an SPICE-based, training-free reconstruction framework for OCT with discontinuous spectra. Representative A-scan and B-scan experiments on multilayer tape and biological tissues suggest that SPICE can visually suppress gap-induced sidelobe artifacts and improve structural interpretability in the tested cases. Quantitative evaluation across 30–75% spectral gaps showed that SPICE improved axial resolution by 30–45% and increased SSIM by 0.15–0.25 compared with gapped FFT. Depth-stratified evaluation under sensitivity roll-off further confirmed that SPICE maintains statistically significant resolution improvement over gapped FFT from the near surface to the roll-off limit. All improvements were statistically significant (*p* < 0.01, paired *t*-test). In addition, the runtime comparison suggests that SPICE has lower computational cost than GAPES in the present MATLAB implementation. Nevertheless, the present results should be interpreted mainly as representative experimental evidence, and systematic quantitative evaluation across broader sample types, gap locations, gap ratios, and SNR conditions remain an important direction for future work.

However, the current study still has certain limitations. First, SPICE is a model-based, training-free estimator that benefits from sparse or structured reflectivity in depth; therefore, its performance may degrade when the depth profile becomes highly dense or when the interferogram SNR is very low. In such cases, sidelobe suppression may trade off with fine-detail preservation. Second, the choice of depth-grid density and stopping tolerance may affect both runtime and peak localization. Extremely large or non-central spectral gaps may also introduce ambiguity, which may require additional priors or multi-band acquisition strategies. Future work will focus on noise-aware variants, adaptive parameter and grid selection, and more comprehensive quantitative evaluation under different gap positions, gap ratios, tissue types, and device-specific spectral non-idealities, to further strengthen the robustness of SPICE for clinical and industrial OCT applications.

## Figures and Tables

**Table 2:** Axial resolution at different depths under a 30% central gap.

Depth (mm)	Gapped FFT (30%, μm)	SPICE FWHM (μm)	*p*-Value
0.3	35.6 ± 0.9	9.8 ± 0.8	<0.001
1.0	42.4 ± 1.5	15.2 ± 1.1	<0.001
1.7	46.2 ± 2.1	21.3 ± 1.5	<0.001

## Data Availability

Data used in this study could be made available upon request.

## Abbreviations

The following abbreviations are used in this manuscript:

OCT	Optical Coherence Tomography
SPICE	Sparse Iterative Covariance-based Estimation
FFT	Fast Fourier Transform
GAPES	Gapped Autocorrelation and Parameter Estimation Spectral Estimation
